# Crystal structures of 2-(benzene­carbo­thio­yloxy)ethyl benzene­carbo­thio­ate and 2-(benzene­carbo­thio­yloxy)ethyl benzoate

**DOI:** 10.1107/S2056989017012701

**Published:** 2017-09-08

**Authors:** Syuto Tanaka, Hyuma Masu, Yuji Sasanuma

**Affiliations:** a Department of Applied Chemistry and Biotechnology, Graduate School and Faculty of Engineering, Chiba University, 1-33 Yayoi-cho, Inage-ku, Chiba 263-8522, Japan; b The Center for Analytical Instrumentation, Chiba University, 1-33 Yayoi-cho, Inage-ku, Chiba 263-8522, Japan

**Keywords:** crystal structure, thio­noester, all-*trans* structure, C⋯S close contact, C—H⋯π inter­action

## Abstract

The title compounds, C_16_H_14_O_2_S_2_ and C_16_H_14_O_3_S, which are monomeric models (models **D** and **E**) for a polythio­noester and a poly(ester-*co*-thio­noester), respectively, crystallize in the space group *P*2_1_/*c* and are isostructural with each other. The mol­ecule in each crystal is located on an inversion centre and has an all-*trans* structure. The asymmetric unit comprises one half-mol­ecule. In the crystal, there are inter­molecular C⋯S contacts [3.391 (3) and 3.308 (3) Å for models **D** and **E**, respectively] and C—H⋯π inter­actions, which form a layer structure parallel to the *bc* plane. The carbonyl and thio­carbonyl groups of the model **E** compound are each disordered over two equivalent sites about the inversion centre with equal occupancies.

## Abstract 2

The title compounds are monomeric models for a polythio­noester and a poly(ester-*co*-thio­noester). The mol­ecules adopt all-*trans* structures with inter­molecular C⋯S close contacts and C—H⋯π inter­actions. Both crystals have almost the same mol­ecular packing in space group *P*2_1_/*c*.

## Chemical context   

Compounds expressed as C_6_H_5_—C(=*X*)—*Y*—CH_2_—CH_2_—*Y*—C(=*X*)—C_6_H_5_ (*X*, *Y* = O or S) can be considered to be monomeric models for polymers, [—C(=*X*)—C_6_H_4_—C(=*X*)—*Y*—CH_2_—CH_2_—*Y*—]_*x*_, namely, *X* = *Y* = O, poly(ethyl­ene terephthalate) (designated herein as polymer **A**); *X* = O and *Y* = S, poly(ethyl­ene di­thio­terephthalate) (polymer **B**); *X* = *Y* = S, poly(ethyl­ene tetra­thio­terephthalate) (polymer **C**); *X* = S and *Y* = O, poly(ethyl­ene di­thio­noterephthalate) (polymer **D**). It is well established that the solution, mechanical and thermal properties of such aromatic polymers are essentially determined by the conformational characteristics of the *Y*—CH_2_—CH_2_—*Y* unit (referred hereafter to as the spacer) and inter­molecular inter­actions between the benzene rings (Sasanuma, 2009 ▸; Sasanuma *et al.*, 2013 ▸). In expectation that replacement of oxygen by sulfur at the *X* or *Y* site would affect the spacer conformation, and π–π and C—H⋯π inter­actions of the benzene rings, and thus lead to variations in the physical properties, we synthesized polymers **B** and **C**, and characterized them by X-ray diffraction, NMR spectroscopy, thermal analyses, mol­ecular orbital calculations and statistical mechanics of the chain mol­ecules (Abe & Sasanuma, 2012 ▸). Herein, the monomeric models for polymers **A**–**D** are termed models **A**–**D**, respectively.

By mol­ecular orbital calculations at the second-order Møller–Plesset perturbation (MP2) level with moderate-size basis sets, we have determined the most stable conformations of the *Y*—CH_2_—CH_2_—*Y* parts of the models and evaluated their free energies relative to that of the all-*trans* form as follows: model **A**, tgt and −1.1 kcal mol^−1^ (Sasanuma, 2009 ▸); model **B**, g^±^tg^∓^ and −3.1 kcal mol^−1^ (Abe & Sasanuma, 2012 ▸); model **C**, g^±^tg^∓^ and −2.1 kcal mol^−1^ (Abe & Sasanuma, 2012 ▸); model **D**, tgt and −1.7 kcal mol^−1^ (this study). We have also predicted that an asymmetric model compound, C_6_H_5_—C(=O)—O—CH_2_—CH_2_—O—C(=S)—C_6_H_5_ (model **E**), would be most stabilized in the tg^±^g^∓^ conformation with a free energy of −1.8 kcal mol^−1^ (this study). However, not all the models and polymers crystallize in the lowest-energy conformations: model (polymer) **A**, ttt (ttt) (Pérez & Brisse, 1976 ▸; Daubeny *et al.*, 1954 ▸); model (polymer) **B**, g^±^tg^∓^ (g^±^tg^∓^) (Deguire & Brisse, 1988 ▸; Abe & Sasanuma, 2012 ▸); model (polymer) **C**, g^±^tg^∓^ (amorphous) (Abe *et al.*, 2011 ▸; Abe & Sasanuma, 2012 ▸). In the crystals, the mol­ecules adopt conformations so as to form inter­molecular inter­actions effectively and minimize the total of intra­molecular and inter­molecular inter­action energies. Inter­estingly, however, models **A**–**C** crystallize in the same spacer conformation as those of the corresponding polymers; therefore, the crystal structure of the model suggests the polymer conformation. This study has aimed to determine crystal structures of the title compounds (models **D** and **E**) to predict the crystal conformations of polymers **D** and **E** on the above hypothesis.
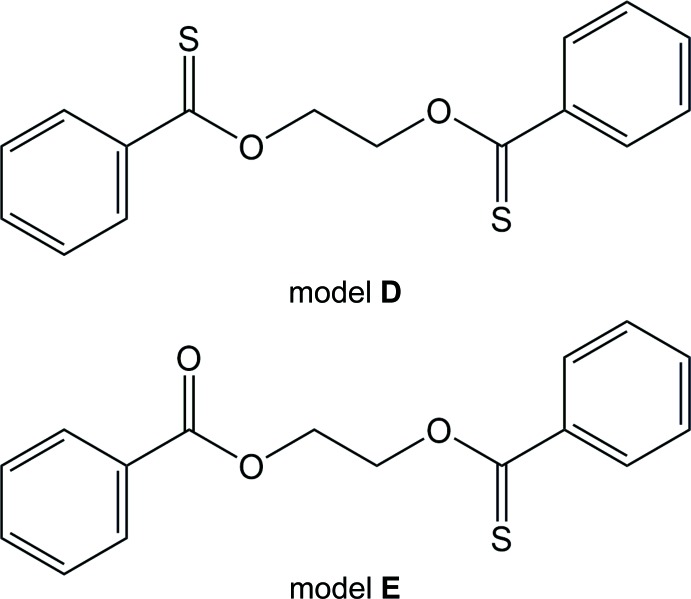


## Structural commentary   

**Figure 1:**
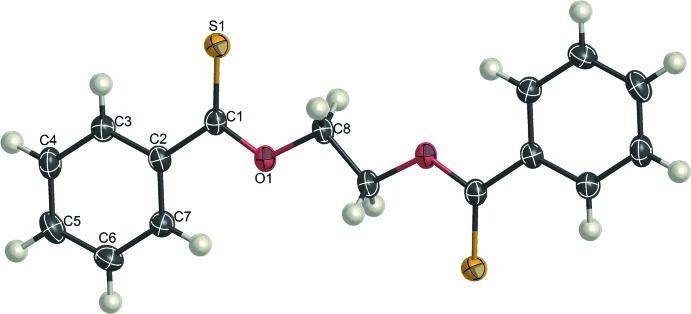
The mol­ecular structure of model **D**, showing atom-labelling scheme. Displacement ellipsoids are drawn at the 50% probability level. The unlabelled atoms are related to the labelled atoms by inversion symmetry (symmetry code: 2 − *x*, 2 − *y*, 2 − *z*,). H atoms are represented by spheres of arbitrary size.

**Figure 2:**
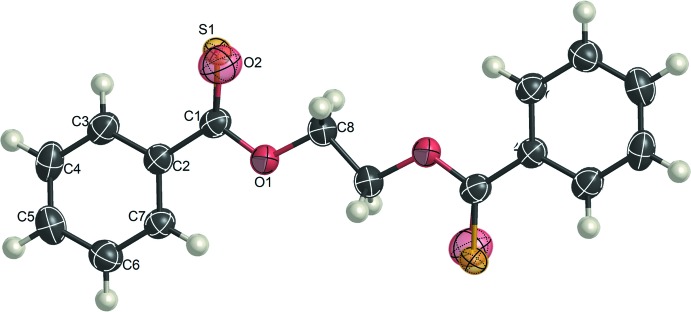
The mol­ecular structure of model **E**, showing atom-labelling scheme. Displacement ellipsoids are drawn at the 50% probability level. The unlabelled atoms are related to the labelled atoms by inversion symmetry (symmetry code: 2 − *x*, 2 − *y*, 2 − *z*,). H atoms are represented by spheres of arbitrary size.

The mol­ecule of model **D** lies on an inversion centre and the asymmetric unit contains one half-mol­ecule. The central O—CH_2_—CH_2_—O unit adopts an all-*trans* conformation (Fig. 1 ▸). The mol­ecule of model **E** is also located on an inversion centre and the O—CH_2_—CH_2_—O bond sequence is in an all-*trans* conformation. Since the mol­ecule has carbonyl and thio­carbonyl groups, the atoms S and O (S1 and O2) are each assumed to be disordered over two equivalent sites about the inversion centre with equal occupancies (Fig. 2 ▸). Consequently, it was proved that all the models (**A**, **D** and **E**) with the O—CH_2_—CH_2_—O spacer crystallize with all-*trans* structures, although models **A**, **D** and **E** in the free state are most stabilized in tgt, tgt, and tg^±^g^∓^ conformations, respectively.

## Supra­molecular features   

**Figure 3:**
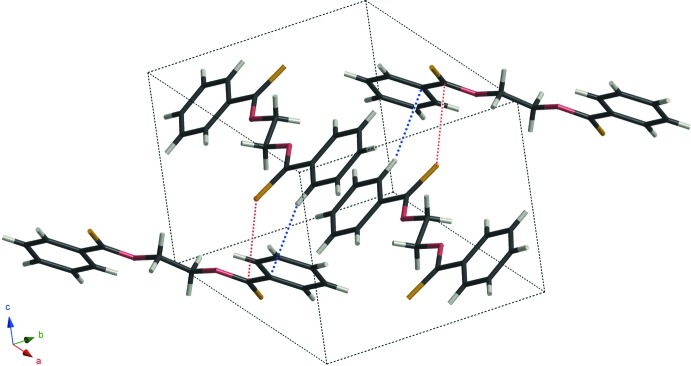
A packing diagram of model **D**, showing inter­molecular C⋯S contacts (red dotted lines) and C—H⋯π inter­actions (blue dotted lines).

The compounds of models **D** and **E** are isotypic and crystallize in the space group *P*2_1_/*c*. There are no classical hydrogen bonds but inter­molecular close contacts between atoms C and S [C1—S1^i^ = 3.391 (3) and 3.308 (3) Å for models **D** and **E**, respectively; symmetry code: (i) *x*, –*y* + 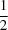, *z* – 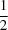]. Both compounds also have C—H⋯π inter­actions (Tables 1 ▸ and 2 ▸) and form layer structures parallel to the *bc* plane *via* these inter­molecular inter­actions (Figs. 3 ▸ and 4 ▸).

### Synthesis and crystallization   

Benzoyl chloride (10.0 ml, 87 mmol) was added dropwise under a nitro­gen atmosphere to ethyl­ene glycol (2.4 ml, 43 mmol) and pyridine (7.0 ml, 87 mmol) placed in a four-necked flask connected to a drying tube filled with calcium chloride, and the mixture was stirred at room temperature overnight. Water was added to the reaction mixture to yield a precipitate, which was collected by filtration, dissolved in chloro­form, washed thrice with 5% aqueous solution of sodium bicarbonate, and dried over anhydrous magnesium sulfate overnight. The liquid phase was separated by filtration and condensed on a rotary evaporator, and the residue was recrystallized from ethanol (15 ml). The white crystallites thus obtained were dried under reduced pressure at room temperature overnight to yield ethane-1,2-diyl dibenzoate (8.3 g, 71%). The synthesized ethane-1,2-diyl dibenzoate (0.10 g, 0.37 mmol) was ground in a mortar and mixed thoroughly with Lawesson’s reagent (0.24 g, 0.59 mmol), and the powder mixture was moved to a 15 ml vial container and placed in a Yuasa PRE-7017R microwave oven. The powder was heated under the following microwave irradiation at 500 W: on for 2.0 min – off for several seconds – on for 1.0 min. The above handling was repeated ten times to obtain the product sufficiently.

The crude product was extracted with chloro­form and condensed under reduced pressure. The residue was dissolved in a mixed solvent of ethyl acetate and *n*-hexane (1:9 *v*/*v*) and subjected to column chromatography. The yellowish fraction (*R*_f_ = 0.5) was collected and condensed, and the residue underwent column chromatography again with a mixed solvent of toluene and *n*-hexane (1:5 *v*/*v*). Two yellow fractions [(1) *R*_f_ = 0.1 and (2) 0.3 − 0.5] were stratified and collected separately. The layer (1) was condensed and recrystallized from ethanol to yield a yellow solid, which was identified as 2-(benzene­carbo­thio­yloxy)ethyl benzoate (model **E**, yield 23%) by ^1^H and ^13^C NMR, and the layer (2) was condensed and dried at room temperature overnight to yield a red solid, which was identified as 2-(benzene­carbo­thio­yloxy)ethyl benzene­carbo­thio­ate (model **D**, yield 0.9%). A small qu­antity of model **D** was dissolved in chloro­form in a thin vial container. The vessel was placed in a larger vial containing a small amount of *n*-hexane, and the outer container was capped. After a week, single crystals were found to be formed in the inner vessel. Single crystals of model **E** were prepared similarly.

## Refinement   

Crystal data, data collection and structure refinement details are summarized in Table 3 ▸. All H atoms were geometrically positioned with C—H = 0.95 and 0.99 Å for the aromatic and methyl­ene groups, respectively, and were refined as riding with *U*_iso_(H) = 1.2 *U*_eq_(C).

## Supplementary Material

Crystal structure: contains datablock(s) General, model_D, model_E. DOI: 10.1107/S2056989017012701/is5479sup1.cif

Structure factors: contains datablock(s) model_D. DOI: 10.1107/S2056989017012701/is5479model_Dsup4.hkl

Structure factors: contains datablock(s) model_E. DOI: 10.1107/S2056989017012701/is5479model_Esup5.hkl

CCDC references: 1572676, 1572675

Additional supporting information:  crystallographic information; 3D view; checkCIF report

## Figures and Tables

**Figure 4:**
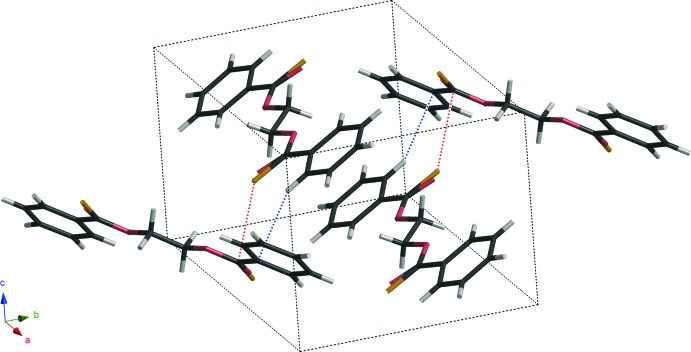
A packing diagram of model **E**, showing inter­molecular C⋯S contacts (red dotted lines) and C—H⋯π inter­actions (blue dotted lines).

**Table 1:** C—H⋯π inter­action geometry (Å, °) for model **D** *Cg*1 is the centroid of the C2–C7 ring.

*D*—H⋯*A*	*D*—H	H⋯*A*	*D*⋯*A*	*D*—H⋯*A*
C3—H3⋯*Cg*1^i^	0.95	2.92	3.721 (3)	143

Symmetry code: (i) *x*, −*y* + 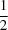, *z* − 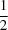.

**Table 2:** C—H⋯π inter­action geometry (Å, °) for model **E** *Cg*1 is the centroid of the C2–C7 ring.

*D*—H⋯*A*	*D*—H	H⋯*A*	*D*⋯*A*	*D*—H⋯*A*
C3—H3⋯*Cg*1^i^	0.95	2.89	3.641 (3)	137

Symmetry code: (i) *x*, −*y* + 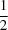, *z* − 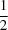.

**Table 3:** Experimental details

	model **D**	model **E**
Crystal data		
Chemical formula	C_16_H_14_O_2_S_2_	C_16_H_14_O_3_S
*M*_r_	302.39	286.33
Crystal system, space group	Monoclinic, *P*2_1_/*c*	Monoclinic, *P*2_1_/*c*
Temperature (K)	173	173
*a*, *b*, *c* (Å)	8.829 (5), 11.680 (7), 7.727 (5)	8.800 (5), 11.403 (6), 7.506 (4)
β (°)	113.475 (10)	113.831 (6)
*V* (Å^3^)	730.9 (8)	689.0 (6)
*Z*	2	2
Radiation type	Mo *K*α	Mo *K*α
μ (mm^−1^)	0.36	0.24
Crystal size (mm)	0.40 × 0.40 × 0.20	0.40 × 0.40 × 0.10

Data collection		
Diffractometer	Bruker APEXII CCD area detector	Bruker APEXII CCD area detector
Absorption correction	Multi-scan (*SADABS*; Sheldrick, 1996 ▸)	Multi-scan (*SADABS*; Sheldrick, 1996 ▸)
*T*_min_, *T*_max_	0.88, 0.93	0.84, 0.98
No. of measured, independent and observed [*I* > 2σ(*I*)] reflections	4017, 1617, 1487	3265, 1523, 1335
*R*_int_	0.014	0.015
(sin θ/λ)_max_ (Å^−1^)	0.648	0.653

Refinement		
*R*[*F* ^2^ > 2σ(*F* ^2^)], *wR*(*F* ^2^), *S*	0.029, 0.080, 1.03	0.038, 0.090, 1.08
No. of reflections	1617	1523
No. of parameters	91	100
H-atom treatment	H-atom parameters constrained	H-atom parameters constrained
Δρ_max_, Δρ_min_ (e Å^−3^)	0.39, −0.20	0.19, −0.16

Computer programs: *APEX2*, *SAINT*, *XSHEL* and *XCIF* (Bruker, 2012 ▸) *SHELXS97* (Sheldrick, 2008 ▸) and *SHELXL2013* (Sheldrick, 2015 ▸).

## supplementary crystallographic information

### 2-(Benzenecarbothioyloxy)ethyl benzenecarbothioate (model_D) Crystal data

**Table:**

C_16_H_14_O_2_S_2_	*F*(000) = 316
*M**_r_* = 302.39	*D*_x_ = 1.374 Mg m^−^^3^
Monoclinic, *P*2_1_/*c*	Mo *K*α radiation, λ = 0.71073 Å
*a* = 8.829 (5) Å	Cell parameters from 2339 reflections
*b* = 11.680 (7) Å	θ = 2.5–27.6°
*c* = 7.727 (5) Å	µ = 0.36 mm^−^^1^
β = 113.475 (10)°	*T* = 173 K
*V* = 730.9 (8) Å^3^	Prismatic, yellow
*Z* = 2	0.40 × 0.40 × 0.20 mm

### 2-(Benzenecarbothioyloxy)ethyl benzenecarbothioate (model_D) Data collection

**Table:**

Bruker APEXII CCD area detector diffractometer	1617 independent reflections
Radiation source: fine-focus sealed tube	1487 reflections with *I* > 2σ(*I*)
Graphite monochromator	*R*_int_ = 0.014
Detector resolution: 8.3333 pixels mm^-1^	θ_max_ = 27.4°, θ_min_ = 2.5°
φ and ω scans	*h* = −10→11
Absorption correction: multi-scan (SADABS; Sheldrick, 1996)	*k* = −13→15
*T*_min_ = 0.88, *T*_max_ = 0.93	*l* = −9→5
4017 measured reflections	

### 2-(Benzenecarbothioyloxy)ethyl benzenecarbothioate (model_D) Refinement

**Table:**

Refinement on *F*^2^	0 restraints
Least-squares matrix: full	Hydrogen site location: inferred from neighbouring sites
*R*[*F*^2^ > 2σ(*F*^2^)] = 0.029	H-atom parameters constrained
*wR*(*F*^2^) = 0.080	*w* = 1/[σ^2^(*F*_o_^2^) + (0.0425*P*)^2^ + 0.2706*P*] where *P* = (*F*_o_^2^ + 2*F*_c_^2^)/3
*S* = 1.03	(Δ/σ)_max_ = 0.001
1617 reflections	Δρ_max_ = 0.39 e Å^−^^3^
91 parameters	Δρ_min_ = −0.20 e Å^−^^3^

### 2-(Benzenecarbothioyloxy)ethyl benzenecarbothioate (model_D) Special details

**Table:**

Experimental. SADABS (Sheldrick 1996)
Geometry. All esds (except the esd in the dihedral angle between two l.s. planes) are estimated using the full covariance matrix. The cell esds are taken into account individually in the estimation of esds in distances, angles and torsion angles; correlations between esds in cell parameters are only used when they are defined by crystal symmetry. An approximate (isotropic) treatment of cell esds is used for estimating esds involving l.s. planes.

### 2-(Benzenecarbothioyloxy)ethyl benzenecarbothioate (model_D) Fractional atomic coordinates and isotropic or equivalent isotropic displacement parameters (Å^2^)

**Table:**

	*x*	*y*	*z*	*U*_iso_*/*U*_eq_	
C1	0.70662 (15)	0.86862 (11)	0.98706 (17)	0.0209 (3)	
C2	0.52641 (15)	0.87852 (10)	0.86705 (17)	0.0205 (3)	
C3	0.41444 (16)	0.80285 (12)	0.89460 (19)	0.0271 (3)	
H3	0.4532	0.7468	0.9916	0.033*	
C4	0.24591 (17)	0.80973 (13)	0.7796 (2)	0.0312 (3)	
H4	0.1705	0.7586	0.7991	0.037*	
C5	0.18837 (16)	0.89124 (13)	0.6367 (2)	0.0309 (3)	
H5	0.0741	0.8947	0.5572	0.037*	
C6	0.29824 (17)	0.96779 (13)	0.6101 (2)	0.0316 (3)	
H6	0.2585	1.024	0.5136	0.038*	
C7	0.46690 (16)	0.96209 (11)	0.72503 (19)	0.0259 (3)	
H7	0.5413	1.0148	0.707	0.031*	
C8	0.97120 (15)	0.94978 (12)	1.04144 (19)	0.0267 (3)	
H8A	1.0184	0.8771	1.0194	0.032*	
H8B	1.0041	0.9599	1.1788	0.032*	
O1	0.79324 (11)	0.95006 (8)	0.94319 (13)	0.0252 (2)	
S1	0.79118 (4)	0.77062 (3)	1.15081 (5)	0.02945 (13)	

### 2-(Benzenecarbothioyloxy)ethyl benzenecarbothioate (model_D) Atomic displacement parameters (Å^2^)

**Table:**

	*U*^11^	*U*^22^	*U*^33^	*U*^12^	*U*^13^	*U*^23^
C1	0.0204 (6)	0.0227 (6)	0.0205 (6)	−0.0032 (5)	0.0091 (5)	−0.0036 (5)
C2	0.0189 (6)	0.0224 (6)	0.0205 (6)	−0.0013 (5)	0.0080 (5)	−0.0036 (5)
C3	0.0232 (6)	0.0300 (7)	0.0265 (7)	−0.0040 (5)	0.0081 (5)	0.0023 (5)
C4	0.0221 (6)	0.0373 (8)	0.0334 (7)	−0.0072 (6)	0.0103 (6)	−0.0024 (6)
C5	0.0191 (6)	0.0376 (8)	0.0318 (7)	0.0020 (5)	0.0057 (5)	−0.0050 (6)
C6	0.0274 (7)	0.0315 (7)	0.0314 (7)	0.0051 (6)	0.0068 (6)	0.0049 (6)
C7	0.0244 (7)	0.0244 (6)	0.0283 (7)	−0.0010 (5)	0.0099 (5)	0.0014 (5)
C8	0.0170 (6)	0.0314 (7)	0.0281 (6)	−0.0041 (5)	0.0051 (5)	0.0030 (5)
O1	0.0174 (4)	0.0269 (5)	0.0282 (5)	−0.0042 (3)	0.0057 (4)	0.0037 (4)
S1	0.02236 (19)	0.0332 (2)	0.0302 (2)	−0.00007 (13)	0.00763 (14)	0.00936 (13)

### 2-(Benzenecarbothioyloxy)ethyl benzenecarbothioate (model_D) Geometric parameters (Å, º)

**Table:**

C1—O1	1.3458 (16)	C5—C6	1.393 (2)
C1—C2	1.4931 (18)	C5—H5	0.95
C1—S1	1.6481 (14)	C6—C7	1.399 (2)
C2—C3	1.4042 (19)	C6—H6	0.95
C2—C7	1.4049 (19)	C7—H7	0.95
C3—C4	1.399 (2)	C8—O1	1.4474 (17)
C3—H3	0.95	C8—C8^i^	1.518 (3)
C4—C5	1.392 (2)	C8—H8A	0.99
C4—H4	0.95	C8—H8B	0.99
			
O1—C1—C2	111.15 (11)	C6—C5—H5	120.0
O1—C1—S1	123.72 (10)	C5—C6—C7	120.22 (13)
C2—C1—S1	125.13 (9)	C5—C6—H6	119.9
C3—C2—C7	119.27 (12)	C7—C6—H6	119.9
C3—C2—C1	119.84 (12)	C6—C7—C2	120.09 (12)
C7—C2—C1	120.89 (11)	C6—C7—H7	120.0
C4—C3—C2	120.17 (13)	C2—C7—H7	120.0
C4—C3—H3	119.9	O1—C8—C8^i^	104.66 (13)
C2—C3—H3	119.9	O1—C8—H8A	110.8
C5—C4—C3	120.21 (13)	C8^i^—C8—H8A	110.8
C5—C4—H4	119.9	O1—C8—H8B	110.8
C3—C4—H4	119.9	C8^i^—C8—H8B	110.8
C4—C5—C6	120.02 (13)	H8A—C8—H8B	108.9
C4—C5—H5	120.0	C1—O1—C8	118.26 (10)

Symmetry code: (i) −*x*+2, −*y*+2, −*z*+2.

### 2-(Benzenecarbothioyloxy)ethyl benzoate (model_E) Crystal data

**Table:**

C_16_H_14_O_3_S	*F*(000) = 300
*M**_r_* = 286.33	*D*_x_ = 1.380 Mg m^−^^3^
Monoclinic, *P*2_1_/*c*	Mo *K*α radiation, λ = 0.71073 Å
*a* = 8.800 (5) Å	Cell parameters from 1991 reflections
*b* = 11.403 (6) Å	θ = 2.5–27.6°
*c* = 7.506 (4) Å	µ = 0.24 mm^−^^1^
β = 113.831 (6)°	*T* = 173 K
*V* = 689.0 (6) Å^3^	Prismatic, yellow
*Z* = 2	0.40 × 0.40 × 0.10 mm

### 2-(Benzenecarbothioyloxy)ethyl benzoate (model_E) Data collection

**Table:**

Bruker APEXII CCD area detector diffractometer	1523 independent reflections
Radiation source: fine-focus sealed tube	1335 reflections with *I* > 2σ(*I*)
Graphite monochromator	*R*_int_ = 0.015
Detector resolution: 8.3333 pixels mm^-1^	θ_max_ = 27.7°, θ_min_ = 2.5°
φ and ω scans	*h* = −11→11
Absorption correction: multi-scan (SADABS; Sheldrick, 1996)	*k* = −6→14
*T*_min_ = 0.84, *T*_max_ = 0.98	*l* = −8→9
3265 measured reflections	

### 2-(Benzenecarbothioyloxy)ethyl benzoate (model_E) Refinement

**Table:**

Refinement on *F*^2^	0 restraints
Least-squares matrix: full	Hydrogen site location: inferred from neighbouring sites
*R*[*F*^2^ > 2σ(*F*^2^)] = 0.038	H-atom parameters constrained
*wR*(*F*^2^) = 0.090	*w* = 1/[σ^2^(*F*_o_^2^) + (0.0288*P*)^2^ + 0.2342*P*] where *P* = (*F*_o_^2^ + 2*F*_c_^2^)/3
*S* = 1.08	(Δ/σ)_max_ = 0.002
1523 reflections	Δρ_max_ = 0.19 e Å^−^^3^
100 parameters	Δρ_min_ = −0.16 e Å^−^^3^

### 2-(Benzenecarbothioyloxy)ethyl benzoate (model_E) Special details

**Table:**

Experimental. SADABS (Sheldrick 1996)
Geometry. All esds (except the esd in the dihedral angle between two l.s. planes) are estimated using the full covariance matrix. The cell esds are taken into account individually in the estimation of esds in distances, angles and torsion angles; correlations between esds in cell parameters are only used when they are defined by crystal symmetry. An approximate (isotropic) treatment of cell esds is used for estimating esds involving l.s. planes.

### 2-(Benzenecarbothioyloxy)ethyl benzoate (model_E) Fractional atomic coordinates and isotropic or equivalent isotropic displacement parameters (Å^2^)

**Table:**

	*x*	*y*	*z*	*U*_iso_*/*U*_eq_	Occ. (<1)
S1	0.78695 (19)	0.76827 (18)	1.1323 (3)	0.0410 (3)	0.5
O2	0.7837 (8)	0.7828 (6)	1.0959 (10)	0.087 (3)	0.5
O1	0.79258 (12)	0.94978 (9)	0.93503 (15)	0.0404 (3)	
C1	0.70747 (18)	0.86474 (13)	0.9769 (2)	0.0372 (3)	
C2	0.52662 (17)	0.87657 (12)	0.86227 (19)	0.0338 (3)	
C3	0.4186 (2)	0.79742 (14)	0.8908 (2)	0.0428 (4)	
H3	0.461	0.7372	0.9857	0.051*	
C4	0.2497 (2)	0.80604 (15)	0.7815 (2)	0.0486 (4)	
H4	0.1762	0.7516	0.8014	0.058*	
C5	0.1873 (2)	0.89288 (16)	0.6442 (3)	0.0492 (4)	
H5	0.0711	0.8978	0.5681	0.059*	
C6	0.2934 (2)	0.97304 (15)	0.6168 (2)	0.0470 (4)	
H6	0.2498	1.0336	0.5229	0.056*	
C7	0.46254 (18)	0.96570 (12)	0.7252 (2)	0.0381 (3)	
H7	0.5351	1.0213	0.7064	0.046*	
C8	0.97020 (18)	0.94635 (14)	1.0342 (2)	0.0440 (4)	
H8A	1.0153	0.8744	0.9999	0.053*	
H8B	1.0047	0.9485	1.1771	0.053*	

### 2-(Benzenecarbothioyloxy)ethyl benzoate (model_E) Atomic displacement parameters (Å^2^)

**Table:**

	*U*^11^	*U*^22^	*U*^33^	*U*^12^	*U*^13^	*U*^23^
S1	0.0345 (5)	0.0398 (5)	0.0452 (6)	0.0040 (4)	0.0125 (4)	0.0086 (5)
O2	0.094 (4)	0.073 (4)	0.086 (5)	−0.007 (3)	0.028 (3)	0.007 (3)
O1	0.0368 (5)	0.0394 (6)	0.0392 (6)	−0.0059 (4)	0.0093 (4)	0.0043 (4)
C1	0.0457 (8)	0.0330 (7)	0.0324 (8)	−0.0042 (6)	0.0153 (6)	−0.0013 (6)
C2	0.0419 (8)	0.0332 (7)	0.0270 (7)	−0.0048 (6)	0.0144 (6)	−0.0058 (5)
C3	0.0536 (9)	0.0417 (8)	0.0333 (8)	−0.0113 (7)	0.0176 (7)	−0.0028 (6)
C4	0.0475 (9)	0.0563 (10)	0.0462 (10)	−0.0191 (8)	0.0233 (7)	−0.0124 (8)
C5	0.0380 (8)	0.0592 (10)	0.0489 (10)	−0.0037 (7)	0.0159 (7)	−0.0100 (8)
C6	0.0445 (8)	0.0470 (9)	0.0464 (10)	0.0036 (7)	0.0151 (7)	0.0021 (7)
C7	0.0427 (8)	0.0343 (7)	0.0380 (8)	−0.0027 (6)	0.0171 (6)	−0.0010 (6)
C8	0.0365 (8)	0.0462 (9)	0.0437 (9)	−0.0044 (7)	0.0103 (6)	0.0033 (7)

### 2-(Benzenecarbothioyloxy)ethyl benzoate (model_E) Geometric parameters (Å, º)

**Table:**

S1—C1	1.549 (3)	C4—H4	0.95
O2—C1	1.279 (6)	C5—C6	1.380 (2)
O1—C1	1.3381 (18)	C5—H5	0.95
O1—C8	1.4349 (19)	C6—C7	1.380 (2)
C1—C2	1.478 (2)	C6—H6	0.95
C2—C3	1.389 (2)	C7—H7	0.95
C2—C7	1.393 (2)	C8—C8^i^	1.501 (3)
C3—C4	1.381 (2)	C8—H8A	0.99
C3—H3	0.95	C8—H8B	0.99
C4—C5	1.374 (3)		
			
C1—O1—C8	117.01 (12)	C4—C5—C6	119.98 (15)
O2—C1—O1	120.5 (3)	C4—C5—H5	120.0
O2—C1—C2	127.6 (3)	C6—C5—H5	120.0
O1—C1—C2	111.73 (13)	C5—C6—C7	120.36 (16)
O1—C1—S1	124.48 (13)	C5—C6—H6	119.8
C2—C1—S1	123.75 (13)	C7—C6—H6	119.8
C3—C2—C7	119.33 (14)	C6—C7—C2	119.85 (14)
C3—C2—C1	119.61 (14)	C6—C7—H7	120.1
C7—C2—C1	121.06 (13)	C2—C7—H7	120.1
C4—C3—C2	120.12 (15)	O1—C8—C8^i^	104.96 (15)
C4—C3—H3	119.9	O1—C8—H8A	110.8
C2—C3—H3	119.9	C8^i^—C8—H8A	110.8
C5—C4—C3	120.33 (15)	O1—C8—H8B	110.8
C5—C4—H4	119.8	C8^i^—C8—H8B	110.8
C3—C4—H4	119.8	H8A—C8—H8B	108.8

Symmetry code: (i) −*x*+2, −*y*+2, −*z*+2.
